# Low-Operating-Temperature NO_2_ Sensor Based on a CeO_2_/ZnO Heterojunction

**DOI:** 10.3390/s21248269

**Published:** 2021-12-10

**Authors:** Kai Sun, Guanghui Zhan, Hande Chen, Shiwei Lin

**Affiliations:** 1 State Key Laboratory of Marine Resource Utilization in South China Sea, School of Materials Science and Engineering, Hainan University, Haikou 570228, China; 19085204210044@hainanu.edu.cn (K.S.); linsw@hainanu.edu.cn (S.L.); 2 Sensor Centre, Hainan Unican Science and Technology Innovation Institute, Haikou 570228, China; chenhande53@gmail.com

**Keywords:** gas sensor, NO_2_ detection, heterojunction, low operating temperature, CeO_2_

## Abstract

CeO_2_/ZnO-heterojunction-nanorod-array-based chemiresistive sensors were studied for their low-operating-temperature and gas-detecting characteristics. Arrays of CeO_2_/ZnO heterojunction nanorods were synthesized using anodic electrodeposition coating followed by hydrothermal treatment. The sensor based on this CeO_2_/ZnO heterojunction demonstrated a much higher sensitivity to NO_2_ at a low operating temperature (120 °C) than the pure-ZnO-based sensor. Moreover, even at room temperature (RT, 25 °C) the CeO_2_/ZnO-heterojunction-based sensor responds linearly and rapidly to NO_2_. This sensor’s reaction to interfering gases was substantially less than that of NO_2_, suggesting exceptional selectivity. Experimental results revealed that the enhanced gas-sensing performance at the low operating temperature of the CeO_2_/ZnO heterojunction due to the built-in field formed after the construction of heterojunctions provides additional carriers for ZnO. Thanks to more carriers in the ZnO conduction band, more oxygen and target gases can be adsorbed. This explains the enhanced gas sensitivity of the CeO_2_/ZnO heterojunction at low operating temperatures.

## 1. Introduction

Nitrogen dioxide (NO_2_) is a harmful gas that threatens human survival [1]. Vehicle exhaust fumes and boiler exhaust emissions are among the principal sources of man-made NO_2_. NO_2_ is a brownish-red, highly reactive gaseous substance that is very harmful to the human body. Even after only a short exposure to nitrogen dioxide, lung function can be impaired [2]. If exposed for a long time, the chance of respiratory infections increases and can lead to permanent organic lesions in the lungs [1,3]. Furthermore, NO_2_ is harmful to the environment and can pollute water, soil, and the atmosphere. Therefore, the rapid and accurate detection of NO_2_ is critical for human health and environmental protection; research on nitrogen dioxide sensors is very important.

In recent research on gas sensors, there is no doubt that sensors based on metal oxide semiconductors (MOSs), graphene [4], polymer nanofibers [5], metal organic frameworks (MOFs) [6], and molecularly imprinted polymers [7] have received the most attention. Compared to other solution strategies, MOS-based sensors provide a cost-effective solution for the rapid deployment of gas detection due to their low power consumption, simplicity of preparation, and ease of integration into electronic devices. Numerous MOS-based chemiresistive gas sensors (ZnO [8,9,10], WO_3_ [11,12], SnO_2_ [13,14], TiO_2_ [15], etc.) have been proven to be used for efficient gas sensing. As a wide bandgap semiconductor-sensitive material, ZnO is a critical component of contemporary gas sensor research due to its cheap cost, high sensitivity, simplicity of manufacture, and miniaturization. However, the disadvantages of MOS-based gas-sensing materials include excessively high operating temperatures and low selectivity, which limit their use in practical engineering applications. The design and synthesis of sensitive materials have been demonstrated in many prior studies to be the most critical element affecting the performance of gas sensors [14]. To promote the practical use of MOS-based chemiresistive sensors, high-sensitivity MOS-based sensors with low operating temperatures are required.

Researchers have synthesised different morphologies of ZnO, including nanorods [16], nanosheets, nanotubes, nanoparticles, nanoplates, nanofilms [17], etc. Different material morphologies have a noticeable influence on the gas-detecting performance of MOS-based gas sensors. The performance of doped ZnO materials has been shown to be improved in several studies. Additionally, several researchers have paired ZnO with other MOSs to make heterojunctions in order to enhance ZnO’s gas-detection performance, which has proven beneficial. Heterojunction structures can be invaluable in adjusting the electrical structure and enabling rich boundary reactions. Han et al. [12] successfully prepared an ordered mesoporous WO_3_/ZnO (OM-WO_3_/ZnO) n–n heterojunction gas sensor. The prepared OM-WO_3_/ZnO sensor significantly improved the response to NO_x_ gas with a shorter response time and lower detection limits. Composite nanostructures of 4 mol% MoO_3_/WO_3_ were reported by Sun et al. [18], which effectively improved the gas sensing performance with lower detection limits (500 ppb).

Cerium oxide (CeO_2_) is a rare-earth oxide catalyst with strong activity, with applications in gas sensors, catalysts, luminescence, and adsorbents [19]. Serpone et al. [20,21] proposed the inter-particle electron transfer (IPET) process, which combines two semiconductors with contrasting redox energy levels in order to improve electron–hole pair separation and carrier concentration. CeO_2_ was found to have sufficient valence and conduction band edges to match ZnO to separate electron–hole pairs [22], thereby supplying more electrons for the redox processes happening at the surface, according to the findings of this study. The construction of heterojunctions from CeO_2_ and ZnO may be employed in this case to enhance the gas sensors.

In this paper, CeO_2_/ZnO heterojunctions were rationally designed and used for NO_2_ gas sensing at low temperatures. The electrodeposition of CeO_2_ onto ZnO nanorods was used in the initial step, followed by the hydrothermal preparation of ZnO nanorods. Materials were studied using several techniques, including X-ray diffractometers (XRDs), ultraviolet–visible absorption spectroscopy (UV–Vis), energy-dispersive X-ray spectroscopy (EDS), field emission scanning selectivity microscopy (FESEM), and transmission electron microscopy (TEM). In comparison to the pure forms of ZnO and CeO_2_, the morphology of the materials did not change much. The sensing performance of the synthesized CeO_2_/ZnO gas sensors was investigated. Compared to the pure ZnO operating temperature of 300 °C, the modified sample exhibits better gas-sensing performance at room temperature (RT), possesses a faster response recovery time, and achieves the best response to NO_2_ at 120 °C, demonstrating the practical potential of the sensor for use at low operating temperatures. 

## 2. Experimental Section

**Figure 1:**
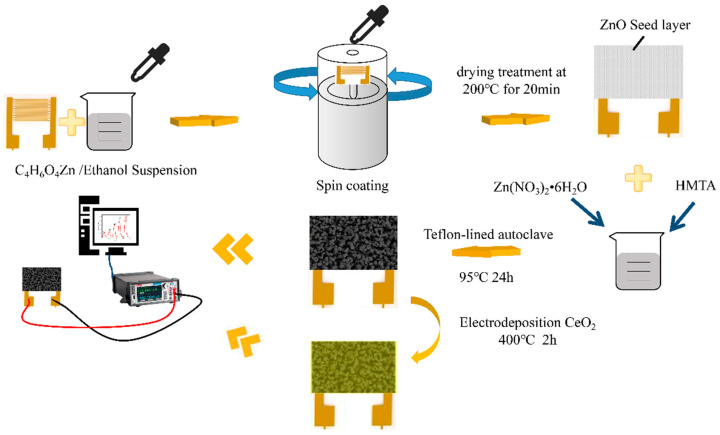
Fabrication and sensing measurements of as-fabricated ZnO-based chemresistive-type sensors.

Unless otherwise mentioned, all compounds used in this study were analytical reagents (AR), and no additional purification was performed. The reagents utilized in this study were obtained from Shanghai Aladdin Biochemical Technology Co. LTD (Shanghai, China). A schematic diagram of the preparation process was shown in Figure 1.

### 2.1. Preparation of ZnO and CeO_2_/ZnO

Preparation of the ZnO seed layer on substrates: Typically, an appropriate amount of Zn(CH_3_COO)_2_·2H_2_O was added to ethanol and vigorously stirred for 20 min as a suspension; in order to prepare the ZnO seed layer on the substrate, a 5.4 mg/mL suspension was spin-coated on Al_2_O_3_ substrates patterned with Au interdigital electrodes (IDEs). Then, drying treatment was carried out at 200 °C for 20 min in a thermostat drier to stabilize the seed layer.

The synthesis process of the pure ZnO nanorods array: The pure ZnO nanorods array was fabricated by a hydrothermal reaction. In 100 mL of deionized water, dispersions of Zn(NO_3_)_2_·6H_2_O (3.56 g) and hexamethylenetetramine (HMTA) (1.67 g) were prepared to provide homogenous solutions A and B, respectively. Then, solution A was gently swirled into solution B. The solution was then transferred to a 50 mL Teflon-lined autoclave vessel with a prepared substrate and maintained at 95 °C for 12 h. Finally, samples were annealed in the air atmosphere for 2 h at a heating rate of 5 °C min^−^^1^ and an annealing temperature of 400 °C.

CeO_2_ is made by grinding cerium dioxide powder. The preparation method of CeO_2_/ZnO is similar to that of the ZnO nanorods array, except with additional anodic electrodeposition coating steps on the ZnO nanorods array in a two-electrode setup at room temperature. IDEs with the ZnO nanorods array were used as the anode, with Pt foil as the cathode. Before anodization, IDEs with the ZnO nanorods array were ultrasonically cleaned in acetone, ethanol, and deionized water, respectively. CeO_2_ was electrochemically grown on a substrate in a 2 M Ce(NO_3_)_3_·6H_2_O solution at a current density of 20 mA cm^−1^ for 1 min, 2 min, 3 min, and 5 min, respectively. Additionally, the corresponding samples were referred to as CeO_2_/ZnO-1, CeO_2_/ZnO-2, CeO_2_/ZnO-3, and CeO_2_/ZnO-4, respectively. Finally, the samples were annealed in the air atmosphere for 2 h at a heating rate of 5 °C min^−^^1^ and an annealing temperature of 400 °C.

### 2.2. Characterization

X-ray diffraction (XRD; Rigaku SmartLab, Osaka, Japan) patterns were used to analyze the phase and crystal structure using a Rigaku SmartLab system with Cu K incident radiation (λ = 1.54056 Å, 20°–80°). Field emission scanning electron microscopy (FESEM; Thermo Scientific Various G4 UC, Brno, Czech Republic), transmission electron microscopy (TEM; Thermo Scientific Talos F200X G2 operated at 200 kV, Brno, Czech Republic), and high-resolution transmission electron microscopy (HRTEM; 200 kV) were used to examine the sample morphologies. TEM attachments were also used to measure the energy-dispersive X-ray spectroscopy (EDS) analysis. An X-ray photoelectron spectrometer was used to analyze the surface chemical elements (XPS; KRATOS Axis Supra, Kyoto, Japan). A UV–Vis spectrophotometer was used to obtain UV–visible diffuse reflectance spectra (UV–Vis; TU-1901, Beijing, China). The electrical signals of the sensors were tested by using a digital source meter (Keithley 2450, Beaverton, OR, USA). Detailed gas-sensitive test methods are available in the Supporting Information.

## 3. Results and Discussion

### 3.1. Morphological and Structural Characteristics

**Figure 2:**
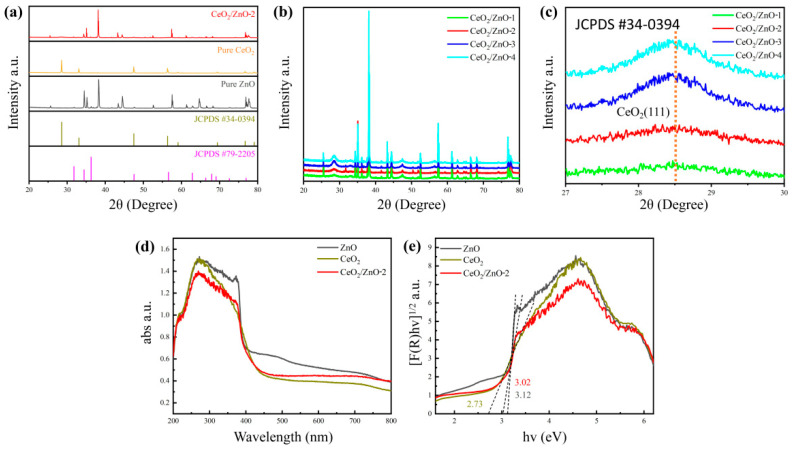
(**a**) XRD patterns within the range of 20° to 80° of pure ZnO, pure CeO_2_, and CeO_2_/ZnO-2. (**b**) XRD patterns within the range of 20° to 80° of CeO_2_/ZnO-1, CeO_2_/ZnO-2, CeO_2_/ZnO-3, and CeO_2_/ZnO-4. (**c**) Enlarged XRD patterns of (111) peak of CeO_2_. (**d**) UV–Vis absorption spectrum of pure ZnO, CeO_2_, and CeO_2_/ZnO-2. (**e**) The bandgap of corresponding samples.

The X-ray diffraction (XRD) patterns of synthesized pure ZnO, pure CeO_2_, and CeO_2_/ZnO-2 nanomaterials are presented in Figure 2a. The typical wurtzite hexagonal peak type of ZnO can be seen from it, and no phase transition from anatase to rutile is observed. Which have corresponded with standard PDF card (JCPDS #79–2205) [23], and could observe the pattern exhibits typical diffraction peaks at 2θ = 31.79°, 34.44°, 36.28°, 47.57°, 56.64°, 62.90°, and 68.00° in all CeO_2_/ZnO composites’ XRD patterns, respectively (Figure 2b) [24,25]. In Figure 2b, all specimens showed the ZnO and CeO_2_ phases, and no other phases were found except for the effect of the substrate. Even at higher Ce loading concentrations, no significant peak shifts were noticed, suggesting that perhaps the Ce ions were not incorporated into the ZnO lattice after a two-hour heat treatment at 400 °C. Zn^2+^ has an ionic radius of 0.74, which is comparable to Ce^4+^ (0.87) but much less than Ce^3+^ (1.01). As a result, Ce^4+^ substitution for Zn^2+^ is possible but not observed in our studies, most likely due to the heat treatment temperature (400 °C) being too low for a solid-state reaction to occur [26,27]. A more pronounced peak of CeO_2_ is observed at 28.5° as the loading concentration of Ce gradually increases (Figure 2c). The absence of CeO_2_ peaks in the samples with less Ce content indicates that the less crystalline CeO_2_ nanoparticles were uniform in size and did not form clusters or granulate [28]. 

In Figure 2d,e, the UV–Vis spectrum pattern of pure ZnO, CeO_2_, and CeO_2_/ZnO-2 is shown. The results show that CeO_2_/ZnO-2 has good absorption of light in the UV wavelength range. According to previous studies, both CeO_2_ and ZnO are direct bandgap semiconductors, which means that electrons from the valence band in both materials can jump directly to the conduction band [29,30]; the Kubelka–Munk equation was used to compute the bandgap (E_g_) of these materials [31]. The (F(R)hv)^1/2^-hv curve and accompanying tangent line were determined using the UV–Vis analysis data, with the intersection of the tangent line and the *x*-axis being the sought bandgap. Based on the results of the calculations it is clear that the E_g_ for ZnO and CeO_2_ is 3.12 and 2.73 eV, respectively. The CeO_2_ bandgap energy value was lower than that reported for bulk cerium oxide (3.15–3.2 eV) [32]. As a result, the CeO_2_/ZnO-2 bandgap was measured to be 3.02 eV, which was somewhat lower than that of as-prepared ZnO (3.12 eV). This finding is significant because it suggests that the CeO_2_/ZnO heterojunction can give more e^−^, which could lead to improved gas-sensing capability.

**Figure 3:**
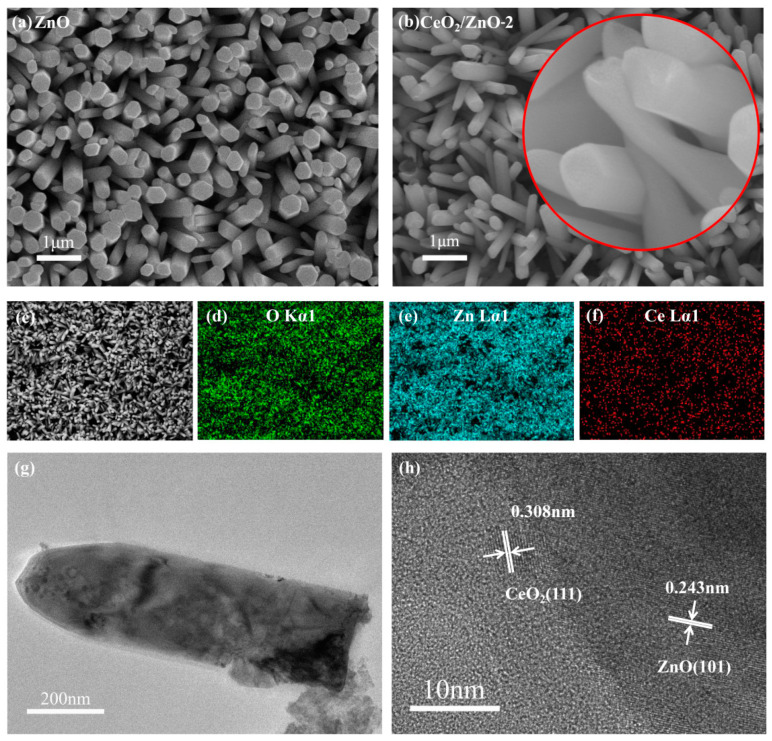
FESEM images of ZnO (**a**) and CeO_2_/ZnO-2 (**b**). (**c**–**f**) EDS images of CeO_2_/ZnO-2; TEM (**g**) and HRTEM (**h**) of CeO_2_/ZnO-2.

The shape of the ZnO nanorods arrays is shown to be affected by the addition of Ce by the FESEM. Figure 3a shows that the pure ZnO nanorod arrays grow uniformly and are randomly oriented in all regions. The nanorods contact each other where they cross, as can be seen. This provides a path for the electrical signal to travel between the nanorods. The ZnO nanorod arrays have a flat surface and the nanorod diameters range from 50 to 100 nm (Figure 3a). Overall, the morphologies of all the CeO_2_/ZnO nanorods arrays were similar (Figure 3b). When the ZnO nanorods are magnified 100,000 times, little nanosheet structures can be clearly observed on their surface (Figure 3b). An EDS spectrum shows that the ZnO nanorods have a Ce-rich composition on their outer layer based on the distribution of these three elements (O, Zn, and Ce) (Figure 3c–f).

TEM and HRTEM images of CeO_2_/ZnO-2 are shown in Figure 3g,h. Pure ZnO has been discovered to have a nanorod-like nanostructure with distinct boundaries. After ZnO and CeO_2_ were compounded no significant changes in nanorod size were observed, and CeO_2_ clusters could be observed on the surface of the nanorods. The lattice fringes of ZnO and CeO_2_ cross and overlap in the composite material, suggesting that a heterojunction structure is formed. The HRTEM image (Figure 3h) shows interplanar spacings of about 0.243 nm and 0.308 nm, which are close to the (101) plane of ZnO and the (111) plane of CeO_2_.

**Figure 4:**
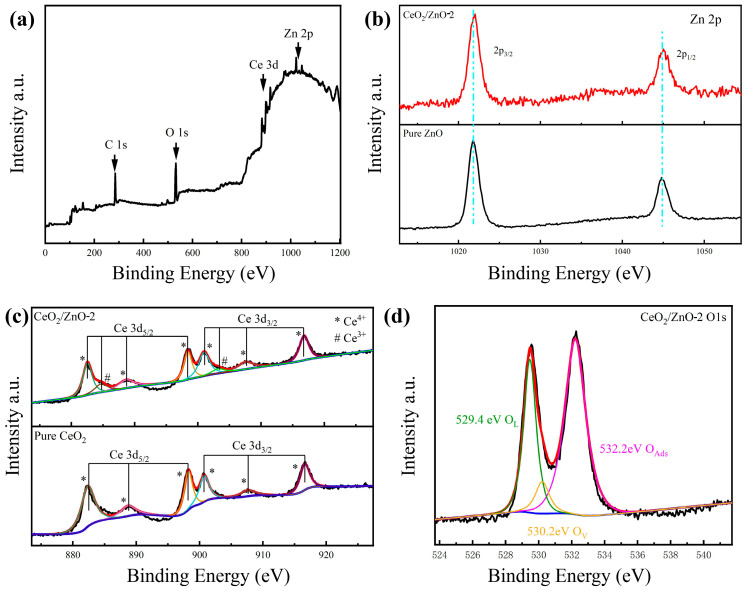
(**a**) Full XPS survey spectra of CeO_2_/ZnO-2. (**b**) Zn 2p peaks of CeO_2_/ZnO-2 composites. (**c**) Ce 3d peaks of CeO_2_/ZnO-2 and pure CeO_2_. (**d**) O1s peaks of CeO_2_/ZnO-2.

The prepared materials’ elemental composition and chemical state were further investigated by utilizing XPS techniques. The investigated XPS spectrum of the prepared sensing material is shown in Figure 4a, which contains mainly C, O, Ce, and Zn peaks. Under the same experimental conditions, the appearance of C1s (284.8 eV) validates the analysis. It is shown in Figure 4b that Zn2p_3/2_ and Zn2p_1/2_ have binding energies of 1021.9 eV and 1045.1 eV, respectively, which are consistent with the values of prepared pure ZnO [33]. Then, we compared the Ce3d energy spectra of CeO_2_/ZnO-2 and pure CeO_2_ in Figure 4d. The peak labeled (*) is for the Ce^4+^ state and the other peaks labeled (#) are characteristic of the Ce^3+^ state, suggesting that the majority of the Ce ions are in the Ce^4+^ state [34]. This suggests that in cerium oxides the Ce ion exists in both Ce^3+^ and Ce^4+^ states and that the corresponding binding energies of these two valence states in the XPS spectra are close. The properties of the Ce 3d final state may be traced to the six peaks at 882.3, 888.7, 898.2, 900.7, 907.6, and 916.6 eV labels generated by three pairs of spin–orbit doublets [35]. Furthermore, two distinct peaks with binding energies of 884.7 and 903.2 eV in the CeO_2_/ZnO composites’ spectra showed the presence of Ce^3+^ surface states in the produced CeO_2_/ZnO composites even though Ce^3+^-containing compounds were not found in the XRD pattern, most likely due to the material’s extremely low concentration of Ce^3+^-containing compounds [36]. O1s spectra are shown in Figure 4c, and the peaks at 529.4, 530.2, and 532.2 eV are attributed to lattice oxygen (O_L_) and oxygen vacancy (O_V_) in ZnO, CeO_2_, and the adsorbed oxygen (O_Ads_) on the sensing materials’ surface [37,38].

### 3.2. Gas-Sensing Properties

For the purpose of comparing the gas-sensing ability of the sensitive materials prepared in this study, we tested pure ZnO, pure CeO_2_, and CeO_2_/ZnO composites prepared by controlled deposition times for 1, 2, 3, and 4 min. The resistance of the pure-CeO_2__-_based gas sensor was far above the range of our existing equipment and its response performance to the gas could not be measured. In subsequent performance tests, pure-ZnO-nanorod-array-based sensors and a series of CeO_2_/ZnO-composite-based sensors were mainly tested. It is important to note that the relative humidity (RH) of the test environment can greatly affect the performance of the sensors, as water in high-humidity air can significantly affect the adsorption of sensitive materials to the target gas or O_2_, and cause changes in the baseline resistance of the sensor, thus affecting the results [39]. Consequently, testing was done in an environment with generally consistent temperatures (25 ± 2 °C) and relative humidity (30 ± 5% RH).

**Figure 5:**
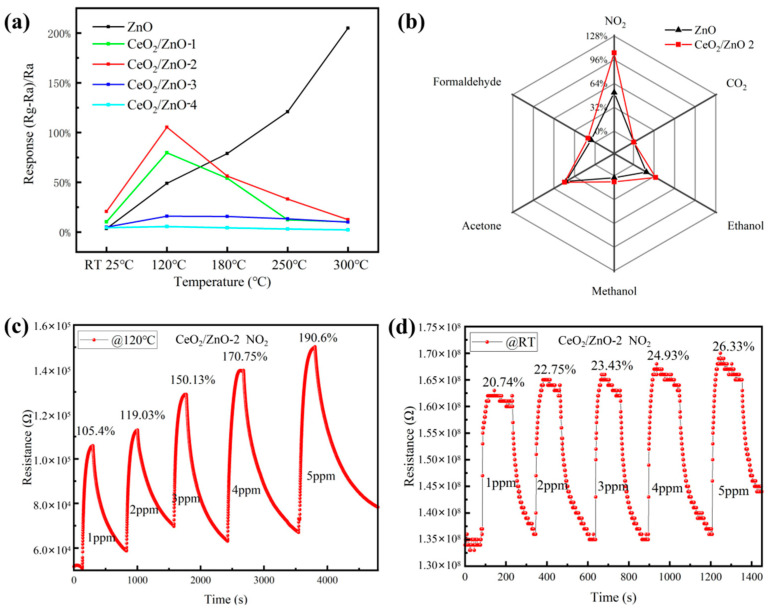
(**a**) Response of sensors as a function of temperature to 1 ppm NO_2_. (**b**) Selectivity of prepared gas sensors (NO_2_: 1 ppm; interference gases: 10 ppm). Resistance of CeO_2_/ZnO-2-based sensor to different NO_2_ concentrations (1 ppm, 2 ppm, 3 ppm, 4 ppm, and 5 ppm) at 120 °C (**c**) and RT (**d**), respectively.

First, the influence of operating temperature on the sensor’s performance was investigated briefly. Due to the limited conditions of the test apparatus, the resistance of the sensitive material under testing needed to be controlled in the range of 0–200 MΩ. However, we found that the resistance of pure CeO_2_ was still too high to exceed the test range. Even when the temperature was increased beyond the heater power range (450 °C), the resistance was still too high. Therefore, all performance tests were carried out on pure ZnO and CeO_2_/ZnO only. In Figure 5a, gas sensitivity tests were carried out over the test temperature range of RT-300 °C for 1 ppm NO_2_, and it was found that all CeO_2_/ZnO samples showed an appreciable response at room temperature relative to the pure ZnO optimum operating temperature at 300 °C and reached optimum operation at 120 °C, followed by a gradual decrease in response intensity with increasing temperature. The best response performance at low temperatures of all samples was obtained for CeO_2_/ZnO-2.

Only when the sensor has good selectivity to the target gas can it be used to distinguish the gas to be measured. Therefore, the gas selectivity of the device is a key indicator for measuring the gas sensitivity of the sensor. Figure 5b shows the results of the selectivity tests of the prepared sensor for different gases. Sensors based on CeO_2_/ZnO-2 composite material and pure ZnO were used to test six different gases under the same test conditions. The gases tested were NO_2_, ethanol, acetone, methanol, formaldehyde, and CO_2_. The concentration of NO_2_ gas was 1ppm, and the concentration of other gases was 10 ppm. It can be seen that the sensor is very selective and that its response is far better to NO_2_ than any other test gas.

The CeO_2_/ZnO-2-based sensor’s response to NO_2_ concentrations ranging from 1 ppm to 5 ppm at 120 °C and 25 °C is shown in Figure 5c,d, respectively. As the concentration rises so does the sensor’s response; even at gas concentrations as low as 1 ppm the CeO_2_/ZnO-2-composite-based sensor has a substantial response. In these two low-temperature experiments, an excellent linear connection between sensor response and target gas concentration was observed (120 °C: R^2^ = 0.986; RT: R^2^ = 0.978).

**Figure 6:**
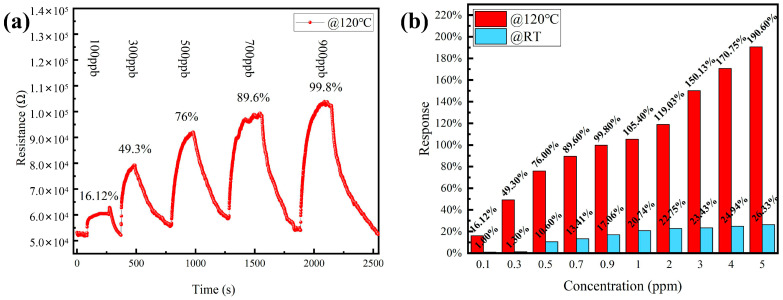
(**a**) Resistance of CeO_2_/ZnO-2-based sensor to different NO_2_ concentrations (100, 300, 500, 700, and 900 ppb) at 120 °C. (**b**) The response of sensors to different NO_2_ concentrations at 120 °C and RT, respectively.

Considering that humans have an odor threshold of 0.5 ppm or less for NO_2_ [3], the gas-sensing performance of the CeO_2_/ZnO-2-based sensor to lower concentrations of NO_2_ at 120 °C was further investigated. Figure 6a shows the change in sensor resistance with gas concentration from 100 ppb to 900 ppb. Figure 6b shows a linear fit of the response to NO_2_ at operating temperatures of 120 °C and 25 °C, respectively. The sensitivity responses of the sensor at 120 °C and RT are shown in Figure 6b.

**Figure 7:**
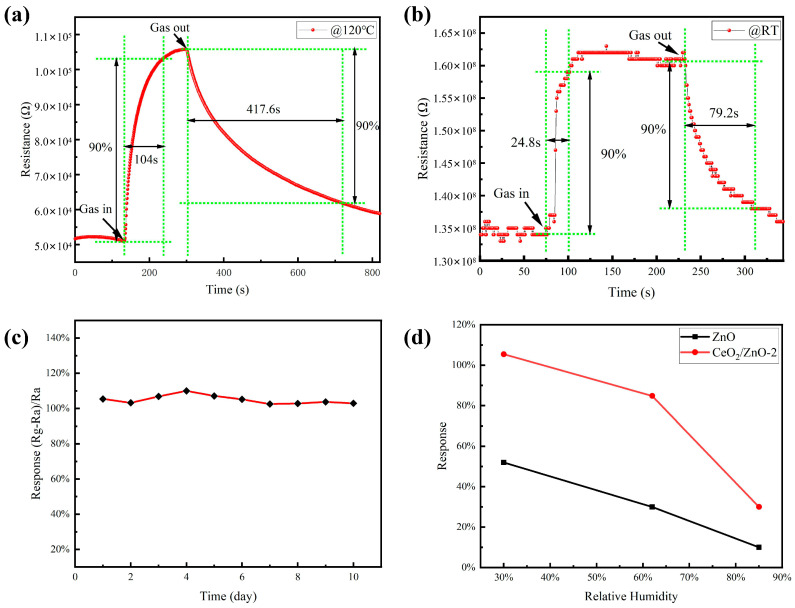
The response/recovery curves of the CeO_2_/ZnO-2-based sensor to 1 ppm NO_2_ at 120 °C (**a**) and RT (**b**), respectively. (**c**) The long-term stability of the CeO_2_/ZnO-2-based sensor. (**d**) The response of sensors on different relative humidity levels (30%, 62%, and 85%).

For sensors that need to be used for practical gas detection, response and recovery time are critical factors in determining if such a sensor is abnormal. The response/recovery curves of the CeO_2_/ZnO-2-based sensor to 1 ppm NO_2_ at RT (25 °C) and 120 °C are shown in Figure 7a,b. Response and recovery time are generally defined as the time required to achieve 90% of the ultimate steady resistance change [40]. The response and recovery time of the CeO_2_/ZnO-2-based sensor at RT can be calculated as 24.8 and 79.2 s, respectively, as shown in Figure 7b. The rapid response/recovery exhibited by the prepared sensor at room temperature test conditions suggests that reversible surface reactions can occur throughout the gas-sensing reaction at room temperature. As shown in Figure 7a, the reaction and recovery times for the CeO_2_/ZnO-2-based sensor at 120 °C can be calculated to be 104 and 417.6 s, respectively. The response/recovery speed is slower than that at room temperature, but the response value has improved substantially. It can be observed that the gas sensitivity of the CeO_2_/ZnO-2-based sensor is greatly improved at low temperatures compared with pure ZnO and that it can respond quickly and sensitively to NO_2_ gas at a lower working temperature. In addition, the long-term stability of the gas-sensitive material was tested at 120 °C, which determines the lifetime of the sensor (Figure 7c). The sensor’s response changed slightly after long-term stability testing (10 days). These experimental results show that sensors based on CeO_2_/ZnO-2 composites provide the possibility of NO_2_ gas detection at low temperatures. The gas-sensitive performance of the sensors was also tested at different relative humidity (RH) levels, with both pure-ZnO- and CeO_2_/ZnO-2-based sensors showing a significant deterioration in gas-sensitive performance as the RH rises at 120 °C (Figure 7d). Overcoming the effect of humidity on gas-sensitive properties is also a problem that needs to be further addressed in subsequent studies.

### 3.3. Sensing Mechanism

Based on the above experimental results and published studies, we propose the following hypothesis for the gas-sensing mechanism and the gas-sensitive enhancement mechanism in this work.

ZnO is a typical n-type MOS that is widely accepted. When used as a gas-sensitive material, it will absorb oxygen in the air. Due to the difference in electronegativity, the adsorbed oxygen will take away electrons from the ZnO surface and form ionic states (O_2_^−^, O^−^, and O^2−^) depending on the operating temperature; <150 °C, 150 to 400 °C, and >400 °C are the regions where O_2_^−^, O^−^, and O^2−^ are the most prevalent [17]. The formulas were expressed as follows:

O_2_ (gas) → O_2_ (adsorbed) (1)

O_2_ (adsorbed) + e^−^ → O_2_^−^ (adsorbed) (2)

O_2_^−^ (adsorbed) + e^−^ → 2O^−^ (adsorbed) (3)

O^−^ (adsorbed) + e^−^ → O^2−^ (adsorbed) (4)

**Figure 8:**
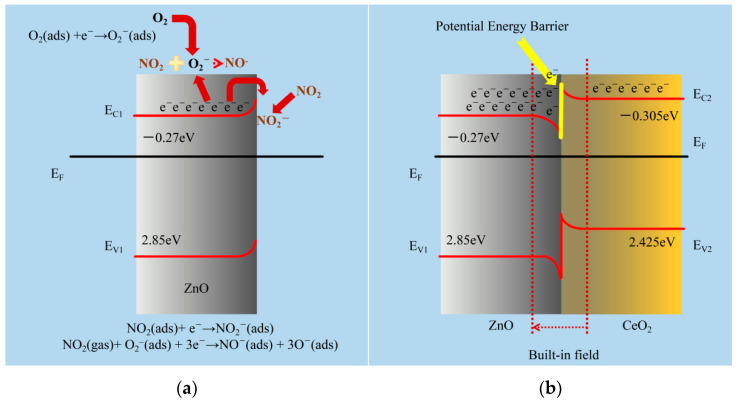
(**a**) The gas-detecting mechanism of the ZnO-based sensors for NO_2_ gas is shown schematically. (**b**) Schematic representations of the proposed increased gas-sensing process for CeO_2_/ZnO-based NO_2_ sensors. E_F_ is the Fermi level; E_C_ and E_V_ are the conduction and valence band edges, respectively (Table S1).

A potential barrier (Δφ) is formed on the surface of the material when oxygen begins to adsorb to its surface. As a result, the ZnO resistance increases. The ZnO’s resistance value will stay constant after the adsorbed oxygen on the material’s surface achieves saturation. This stable resistance value is called the baseline resistance. When ZnO is in contact with an oxidizing gas such as NO_2_, the gas molecules will react with oxygen ions and the ZnO surface to obtain electrons, which causes the thickness of the space charge layer on the ZnO surface to increase, Δφ becomes larger, and the surface resistance increases (Figure 8a). The formulas were expressed as follows:

NO_2_ (gas) → NO_2_ (adsorbed) (5)

NO_2_ (adsorbed) + e^−^ → NO_2_^−^ (adsorbed) (6)

NO_2_ (gas) + O^2−^ (adsorbed) + 3e^−^ → NO^−^ (adsorbed) + 3O^−^ (adsorbed) (7)

Following the manufacturing of the heterojunction, an internal built-in field from the CeO_2_ zone to the ZnO zone will develop at the interface due to charge accumulation, as shown in Figure 8b [41,42], while decreasing the likelihood of electrons in the ZnO conduction band transferring to CeO_2_ owing to the existence of a potential barrier at the interface [43,44]. With the effect of the internal built-in field and the potential barrier, the ZnO side of the heterojunction accumulates more electrons, increasing the carrier concentration significantly. This enables the material to absorb more oxygen ions and target gases without the need for additional excitation conditions. 

## 4. Conclusions

In this work, we successfully synthesized a NO_2_ gas sensor based on a CeO_2_/ZnO heterojunction by using simple electrodeposition followed by the hydrothermal method. The heterostructure consists of an array of ZnO nanorods and tiny CeO_2_ nanocrystals. At low operating temperatures, CeO_2_ coupling significantly improves the sensing ability of ZnO for NO_2_. This is mainly due to the large increase in carrier concentration of the sensing material caused by the built-in field formed after the construction of the heterojunction. In addition, we found that compared to other samples, CeO_2_/ZnO-2 heterojunctions are the best for optimizing gas-sensitive properties when used as gas-sensitive materials. In summary, a series of CeO_2_/ZnO n–n type heterostructured gas-sensing materials were synthesized by electrodeposition with low operating temperatures and good stability at low temperatures (RT, 120 °C), leading us to explore them as promising NO_2_-sensitive materials.

## Data Availability

Not applicable.

## Supplementary Materials

The following are available online at https://www.mdpi.com/article/10.3390/s21248269/s1, Table S1: The main values for calculating CB and VB potentials of ZnO and CeO_2_.
