# Supplementary material for: Low-Operating-Temperature NO2 Sensor Based on a CeO2/ZnO Heterojunction
Source: Sensors (Basel). 2021 Dec 10;21(24):8269. doi: 10.3390/s21248269 (PMC8705860; doi:10.3390/s21248269)
Supplement: Supplementary file 1 [file sensors-21-08269-s001.zip › sensors-1493257-supplementary.pdf]

Low-Operating-Temperature NO_2 Sensor based on CeO_2/ZnO Heterojunction

Kai Sun [1], Guanghui Zhan [1,*], Hande Chen [2], Shiwei Lin [1]

[1] State Key Laboratory of Marine Resource Utilization in South China Sea, School of Materials Science and Engineering, Hainan University, Haikou 570228, China; 19085204210044@hainanu.edu.cn (K.S.); linsw@hainanu.edu.cn (S.L.)
[2] Sensor Centre, Hainan Unican Science and Technology Innovation Institute, Haikou 570228, China; chenhande53@gmail.com
* Correspondence: ghzhan@hainanu.edu.cn

Gas sensing measurement

Sensors need to be aged for 24 hours before testing, this helps to minimize materials defects and increase sensor stability. Air is utilized as a background gas, a conical flask (1000ml) was filled with air and a sensor was put in it to measure the resistance. Then the sensor was put in a conical flask with a predetermined concentration of target gas, and once the sensor resistance attained a new constant value, the sensor was then inserted into an identical size conical flask full of air to recover. Electric signals were measured using a Keithley 2450 SourceMeter. Rg / Ra (for oxidizing gas) is the ratio of the sensor's air resistance to the target gas's electrical resistance. Response and recovery times are defined as the amount of time it takes for the sensor resistance to achieve 90% of its response value after exposure to the target gas and 10% of its response value after the target gas has been withdrawn.

For semiconductors, CB and VB can be calculated according to the following empirical equations:

$$ECB=\chi\text{-}Ee - 0.5Eg \quad (1)$$

$$EVB= Eg + ECB \quad (2)$$

Where E_{CB} is the CB edge potential; E_{VB} is the VB edge potential; χ is the electronegativity of the semiconductor; E_e is the energy of free electrons on the hydrogen scale (about 4.5 eV); E_g is the bandgap energy of the semiconductor. Therefore, the main values for calculating CB and VB potentials of ZnO and CeO_2 are listed in Table 1. The results showed that the CB and VB of ZnO lie below the CB and VB of CeO_2, respectively.

Table S1. The main values for calculating CB and VB potentials of ZnO and CeO_2.

	χ	E_g (eV)	E_{CB} (eV)	E_{VB} (eV)
ZnO	5.79	3.12	-0.27	2.85
CeO_2	5.56	2.73	-0.305	2.425
